# Development of an HIV-1 Subtype Panel in China: Isolation and Characterization of 30 HIV-1 Primary Strains Circulating in China

**DOI:** 10.1371/journal.pone.0127696

**Published:** 2015-05-27

**Authors:** Jingwan Han, Siyang Liu, Wei Guo, Zuoyi Bao, Xiaolin Wang, Lin Li, Yongjian Liu, Daomin Zhuang, Hanping Li, Lei Jia, Tao Gui, Hongshuai Sui, Tianyi Li, Jingyun Li

**Affiliations:** Department of AIDS Research, State Key Laboratory of Pathogen and Biosecurity, Beijing Institute of Microbiology and Epidemiology, 0007, Beijing, P.R. China; Chinese Academy of Sciences, Wuhan Institute of Virology, CHINA

## Abstract

**Background:**

The complex epidemic and significant diversity of HIV-1 strains in China pose serious challenges for surveillance and diagnostic assays, vaccine development and clinical management. There is a lack of HIV-1 isolates in current canonical HIV-1 subtype panels that can represent HIV-1 diversity in China; an HIV-1 subtype panel for China is urgently needed.

**Methods:**

Blood samples were collected from HIV-1 infected patients participating in the drug-resistance surveillance program in China. The samples were isolated, cultured and stored as neat culture supernatant. The HIV-1 isolates were fully characterized. The panel was used to compare 2 viral load assays and 2 p24 assays as the examples of how this panel could be used.

**Results:**

An HIV-1 subtype panel for China composed of 30 HIV-1 primary strains of four subtypes (B [including Thai-B], CRF01_AE, CRF07_BC and G) was established. The samples were isolated and cultured to a high-titer (10^6^-10^9^ copies/ml)/high-volume (40ml). The HIV-1 isolates were fully characterized by the final viral load, p24 concentration, *gag-pol* and *env*C2V3 sequencing, co-receptor prediction, determination of the four amino acids at the tip of the *env* V3-loop, glycosylation sites in the V3 loop and the drug-resistance mutations. The comparison of two p24 assays and two viral load assays on the isolates illustrated how this panel may be used for the evaluation of diagnostic assay performance. The Pearson value between p24 assays were 0.938. The viral load results showed excellent concordance and agreement for samples of Thai-B, but lower correlations for samples of CRF01_AE.

**Conclusion:**

The current panel of 30 HIV-1 isolates served as a basis for the development of a comprehensive panel of fully characterized viral isolates, which could reflect the current dynamic and complex HIV-1 epidemic in China. This panel will be available to support HIV-1 research, assay evaluation, vaccine and drug development.

## Introduction

It has been reported that the number of people living with HIV increased to 35 million (33.2 million–37.2 million) by the end of 2013 according to the United National Programme on HIV and AIDS (UNAIDS) [1]. Currently, the major source of the global pandemic is divided into four groups: M (Major), O (Outlier), N (Non-M and Non-O) and P. The M group includes nine major subtypes (i.e., A-D, F-H, J, and K), over 72 circulating recombinant forms (CRF), and numerous unique recombinant forms (URF). Their rapid evolution and comprehensive diversity make them a serious challenge for the maintenance of a reliable serological and nucleic acid test for blood screening, epidemiological surveillance, diagnosis, and clinical management of the infected persons. Differences between subtypes in HIV-1 RNA quantitation and determination, serological diagnostic and screening variations have been noted [2, 3]. Many HIV-1 nucleic acid tests or serological assays designed and tested based on subtype B virus have demonstrated an underestimation of some non-B subtypes [4–6]. Differences between subtypes in currently commercially available quantitative viral load tests have been reported [7–9]. Furthermore, viral diversity may also impact responses to antiretroviral therapies and vaccines [10–12]. Under these circumstances, a set of fully characterized viruses from HIV infections are needed to better represent current global HIV diversity and help control the worldwide HIV-1 epidemic through support of epidemiological testing, vaccines, and therapeutic efforts [13].

HIV-1 subtype B (Thailand’s variant of subtype B included), CRF01_AE, CRF07_BC and CRF08_BC are the major subtypes of HIV-1 strains circulating in China [14]. HIV-1 strains of subtype B from Thailand were transmitted to Yunnan province in the early 1990s. Strains of subtype C from India were transmitted to Yunnan soon after 1993 and these two subtypes caused the first wave of an HIV-1 epidemic in China among intravenous drug users (IDUs) in Yunnan province [15]. CRF07_BC and CRF08_BC were generated from subtypes B and C, and most likely originated in Yunnan [16–18]. CRF08_BC spread eastward to the southern coastal provinces around 1990 [16, 18], while CRF07_BC spread to northwestern China along a drug traffic route around 1993 [15, 18, 19]. Meanwhile, unregulated and unsanitary commercial plasma collection between the years 1992 and 1995 in Henan caused an HIV-1 subtype Thai-B infection epidemic in central China [20–22]. On the other hand, since HIV-1 CRF01_AE was first found among heterosexuals and IDUs in southern China around 1996–1997, this subtype of the virus spread through sexual routes to other provinces quickly and broadly [23–25]. Several new circulating recombinant forms generated from the subtypes circulating in China were found recently [26–32], such as CRF55_01B, which was a recombinant of CRF01_AE and Thai-B and was discovered from men having sex with men (MSM) in the year 2013 [26]; CRF57_BC, which was a recombinant of Thai-B and India C and was discovered in western Yunnan in China in 2014 [27]; and CRF61_BC, which was comprised of CRF07_BC and CRF08_BC and was identified in the heterosexual population in two different regions in China [29]. The diversity of HIV-1 is extensive in China.

In the U.S., a pilot program called the NIAID External Quality Assurance Program Oversight Laboratory (EQAPOL) established by the National Institutes of Health/National Institute of Allergy and Infectious Diseases (NIH/NIAID) in collaboration with other institutes throughout the world developed an HIV subtype panel that encompassed the genetic and geographic diversity currently present worldwide [13, 33]. This program has collected over 100 viral samples, but only 7 of them were isolated from HIV-1 infections in China [13].

To establish an HIV-1 subtype panel that represents the diversity of HIV-1 strains circulating in China, we collected 99 plasma specimens from HIV-1 infected individuals in 10 provinces of China through the HIV-1 drug-resistance surveillance program. Finally, we successfully obtained 30 HIV-1 isolates of different subtypes. The HIV-1 p24 antigen concentrations and viral loads of the isolates were evaluated. We also sequenced the three major structural genes: *gag*, *pol* and *env*C2V3 for subtype analysis, prediction of co-receptor usage, the four amino acids at the tip of the V3-loop and the glycosylation sites on *env*V3 sequence, as well as the drug-resistance mutations.

## Materials and Methods

### Patients and samples

Blood samples with the basic personal information of each patient were collected from HIV infected individuals who were participants in the HIV-1 drug-resistance surveillance program in 10 provinces of China (Guangxi, Guangdong, Sichuan, Henan, Xinjiang, Ningxia, Shanghai, Shandong, Beijing and Zhejiang). 10 ml of anticoagulated whole blood was obtained from each patient for virus isolation and expansion. The basic personal information of the patients showed that all the main routes of HIV-1 transmission were included among the cases (i.e., homosexual sex, heterosexual sex, blood donation, blood transfusion, intravenous drug using and occupational exposure).

All the samples were identified by HIV-1 antibody enzyme linked immunosorbent assay (ELISA) screening, HIV-1 p24 antigen testing and the Western blot to confirm the infection of HIV-1.

### Isolation and expansion of primary HIV-1 strains

Peripheral blood mononuclear cells (PBMCs) from the blood samples were separated immediately after the collection, and they were co-cultured with PBMCs from healthy blood donors to separate and expand the virus in a three-step process: isolation, small-scale culture and large-scale culture.

For isolation, the healthy PBMCs were isolated from the blood of more than eight healthy blood donors by HISTOPAQUE-1077 (SIGMA). The isolated PBMCs were cultured in RPMI-1640 medium with 10% fetal bovine serum (FBS), 100 U/ml penicillin and 100 μg /ml streptomycin. The healthy PBMCs were stimulated by 5 μg/ml phytohemagglutinin (PHA) and 10 IU/ml interleukin-2 (IL-2) for three days. Then, the infected PBMCs were separated from the patients’ blood in the same way and cultured in RPMI1640 medium with FBS, penicillin, streptomycin and IL-2 but without PHA. The patient's PBMCs and activated healthy donor PBMCs were centrifuged at 250×*g* for 5 minutes, and then the PBMCs were re-suspended in RPMI-1640 medium to 2×10^6^ cells/ml respectively. Infected and healthy PBMCs were mixed at a volume ratio of 1:1 into 24-well plates and were then co-cultured in a cell incubator containing 5% CO_2_ at 37°C. A total of 100 μl of supernatant was sampled every seven days to measure the concentration of p24 antigen to observe the growth of every HIV-1 strain, and one half of the initial volume of the cell supernatant was replaced by healthy donor cells to maintain a stable environment for virus growth. The cultures were continued for up to 28 days or until p24 results were greater than 2 pg/ml.

For small-scale culture, 4 ml of 2×10^6^ cells/ml PBMCs from healthy donors were inoculated with 1 ml HIV-1 cell culture supernatant sampled at the end of the isolation, and the viruses were cultured for another 7 days until the 35^th^ day or until the p24 results were greater than 10 pg/ml.

For large scale culture, 1 ml of cell supernatant from the small-scale culture were put into 39 ml of 1×10^6^cells/ml PBMCs, and cultured for another 7 days or until the p24 antigen results were greater than 25 pg/ml.

The cell suspensions were centrifuged at 800×*g* for 5 minutes and the supernatant was aliquoted into 1.5 ml centrifuge tubes at 1 ml/tube and stored in liquid nitrogen (-196°C).

### HIV-1 *gag*, *pol*, *env*C2V3 and full-length genome amplification and sequencing

The full-length of *gag* and *pol* were amplified and sequenced and then spliced together as a complete genetic region for subtyping. The C2V3 region of *env* was also amplified and sequenced.

Viral RNA from the culture supernatant was extracted with a QIAamp Viral RNA Mini Kit and served as the template for amplification. The 1,502 bp full length *gag* gene (HXB2: 790–2292), 3,011 bp full length *pol* gene (HXB2: 2,085–5,096) and 520 bp *env*C2V3 (HXB2: 7021–7541) fragments were amplified according to reference [34]. The full-length *gag* and *pol* genes were connected together as a complete genetic region (HXB2: 790–5,096) for subtyping.

### Phylogenetic analysis and subtype identification

In order to identify the subtypes of the isolated strains, we regarded the *gag-pol* fragment as the target sequence. A neighbor-joining tree of these target sequences and reference strains (subtypes A–D, F–H, J, K and most kinds of circulating recombinant forms were included) was constructed by MEGA5.1 with bootstrap 1000. Virus samples that were unidentified in the *gag-pol* fragment were uploaded to REGA (http://jose.med.kuleuven.ac.be/genotypetool/html/subtypinghiv.html) and NCBI HIV Subtyping. Recombinants and incomplete *gag-pol* sequences of the recombinants were analyzed using the jumping profile hidden Markov model (jpHMM) tool provided at www.hiv.lanl.gov (http://jphmm.gobics.de/).

In order to identify the diversity of CRF01_AE isolates in our program, we performed phylogenetic analysis for the CRF01_AE isolates in our panel in particular by using the full-length sequences of the gene *pol* from the isolates, together with the HIV-1 CRF01_AE sequences used in Yi Feng and Xiang He’s study [35]. A neighbor-joining tree was constructed by MEGA5.1 with bootstrap 1000.

### The prediction of co-receptor usage, the four amino acids at the tip of the V3-loop and the glycosylation sites by *env*V3

The genotype prediction of co-receptor usage of the HIV-1 isolates based on the *env*V3 sequence information was predicted with two online software programs: a Web-based genotypic algorithm—WebPSSM (http://intra.mullins.microbiol.washington.edu/webpssm/) and Geno2Pheno (G2P) prediction, which is based on the bioinformatics prediction tool that uses the V3 sequence plus additional host-specific features to select false positive rates (FPR) at 1%, 2.5%, 5%, 15%, and 20%. The FPR represents the percentage of all samples that are X4-incapable will be predicted as X4-capable viruses, and FPR at 2.5% was used in this program; http://coreceptor.geno2pheno.org/index.php. We translated the nucleotide sequence of the V3 loop into the corresponding amino acid sequence using software Gene Cutter provided by the Los Alamos HIV database (http://www.hiv.lanl.gov/content/sequence/GENE_CUTTER/cutter.html), and the top four amino acids at the tip of the V3-loop of each HIV-1 isolate. In addition, the glycosylation sites on the V3-loop were predicted by online prediction software N-GlycoSite (http://www.hiv.lanl.gov/content/sequence/GLYCOSITE/glycosite.html).

### The determination of viral drug resistance mutations

We submitted the full-length *pol* gene (2,085 bp–5,096 bp) to the Stanford University Network HIV-1 database (http://hivdb.stanfrod.edu/hiv/) after amplification and sequencing to determine drug resistance mutations and tolerance of various antiviral drugs of the HIV-1 isolates.

### Measurement of HIV-1 isolate p24 concentration and viral load after isolation and expansion

We detected p24 antigen and measured the viral load to evaluate the correlation and agreement between the two indicators.

P24 antigen was quantified using a BioMerieux VIDAS HIV p24 II assay and one domestic p24 test assay. For the samples that were beyond the upper limits (400 pg/ml for the VIDAS HIV p24 II assay and 80 pg/ml for the domestic p24 test assay), the RPMI-1640 medium with 10% FBS was used for dilution in the performance of both of the assays.

The viral loads of all the 30 samples in the panel were measured using two commercial kits according to the manufacturers’ instructions: the automated Roche Cobas AmpliPrep/Cobas TaqMan HIV-1 test version 2.0 assay (CAP/CTM v2.0 assay) and Nuclisens HIV-1 EasyQ version 2.0 assay (EasyQ v2.0 assay). For the samples that were beyond the upper limits (5,757,126 IU/ml for EasyQ v2.0 assay and 17,000,000 IU/ml for the CAP/CTM v2.0 assay [36]), HIV-1-negative plasma was used to dilute the culture supernatant of the isolates in the performance of the two assays.

To compare the performance of the CAP/CTM v2.0 assay with that of the EasyQ v2.0 assay, all HIV-1 viral load values (expressed as copies/ml) were converted to IU/ml, and then into log IU/ml values for statistical analysis [36]. The correlation coefficient (R) of Pearson test was used to assess the strength of the linear association and the Bland-Altman method was used to assess the level of agreement between the paired measurements [37].

### Statistical analysis

The mean differences and standard deviations (SD) were calculated using SPSS 22.0 software to compare the performance of the 2 viral load measurement assays. The Pearson correlation coefficient (R) was used to assess the strength of the linear association between the log-transformed levels in the positive samples measured by the two viral load assays and between the p24 concentration values tested by the two p24 assays. The Bland-Altman method was used to assess the level of agreement between the paired measurements using MedCalc 14.8.1.0 software [37]. The linear association between the p24 concentrations measured by the BioMerieux VIDAS HIV p24 II assay and the HIV-1 viral loads measured by a Roche Cobas AmpliPrep/Cobas TaqMan HIV-1 test version 2.0 assay (CAP/CTM v2.0 assay) had also been evaluated.

### Ethical statement

The study has been ethically approved by the Ethical Board of the Beijing Institute of Microbiology and Epidemiology. Blood samples were collected from the patients participated in the drug-resistance surveillance program in China. The written informed consents were obtained from all the participants and the data were analyzed anonymously. The informed consent process has also been approved by the Ethical Board of the Beijing Institute of Microbiology and Epidemiology.

## Results

### Virus isolation and expansion

After seven times of isolation and expansion in different years, the p24 concentrations of 30 isolates were greater than 25 pg/ml. As for the 30 HIV-1 primary strains that were isolated and expanded successfully, strains from each of the 10 provinces in China still existed; i.e., Guangxi (n = 4), Guangdong (n = 5), Sichuan (n = 1), Henan (n = 11), Xinjiang (n = 1), Ningxia (n = 1), Shanghai (n = 1), Shandong (n = 4), Beijing (n = 1) and Zhejiang (n = 1). The basic personal information of the cases is summarized in Table 1.

**Table 1:** Basic information on the 30 HIV-1 samples.

Sample ID	Gender^a^	Infection route	Area of origin	Year	Treatment^b^
HN2002024	M	Blood transfusion	Henan	2002	Y
GX2005002	M	Heterosexual sex	Guangxi	2005	N
GX2005016	M	Blood transfusion	Guangxi	2005	N
GD2005003	M	Blood transfusion	Guangdong	2005	Y
GD2005004	M	Heterosexual sex	Guangdong	2005	Y
GD2005006	M	Heterosexual sex	Guangdong	2005	N
GD2005025	M	intravenous drug using	Guangdong	2005	N
GD2005028	F	Blood transfusion	Guangdong	2005	N
NX2005012	M	Blood transfusion	Ningxia	2005	N
GX2006183	M	Unclear	Guangxi	2006	Y
GX2006185	F	Heterosexual sex	Guangxi	2006	Y
BJ2006001	M	Heterosexual sex	Beijing	2006	Y
SD2006001	M	Blood transfusion	Shandong	2006	Y
ZJ2006001	F	Blood transfusion	Zhejiang	2006	Y
SH2007052	M	Heterosexual sex	Shanghai	2007	Unknown
SC2009001	M	Occupational Exposure	Sichuan	2009	Y
SX2010001	M	Blood transfusion	Shanxi	2010	Y
SD2010001	M	Homosexual sex	Shandong	2010	N
HN2010001	M	Blood donation	Henan	2010	N
HN2010002	M	Blood donation	Henan	2010	N
BJ2010001	M	Homosexual sex	Beijing	2010	N
HN2010003	F	Blood transfusion	Henan	2010	N
HN2010004	F	Blood donation	Henan	2010	N
BJ2010002	M	Homosexual sex	Beijing	2010	N
XJ2010001	M	Homosexual sex	Xinjiang	2010	N
HN2010005	F	Blood donation	Henan	2010	N
BJ2010003	M	Homosexual sex	Beijing	2010	N
SD2013001	M	Homosexual sex	Shandong	2013	N
SD2013005	M	Homosexual sex	Shandong	2013	N
SD2013008	M	Homosexual sex	Shandong	2013	N

^a^: “M” represents for male and “F” represents for female.

^b^: “Y” represents for treated and “N” represents for treated naive.

### Phylogenetic analysis and subtype identification

**Fig 1:**
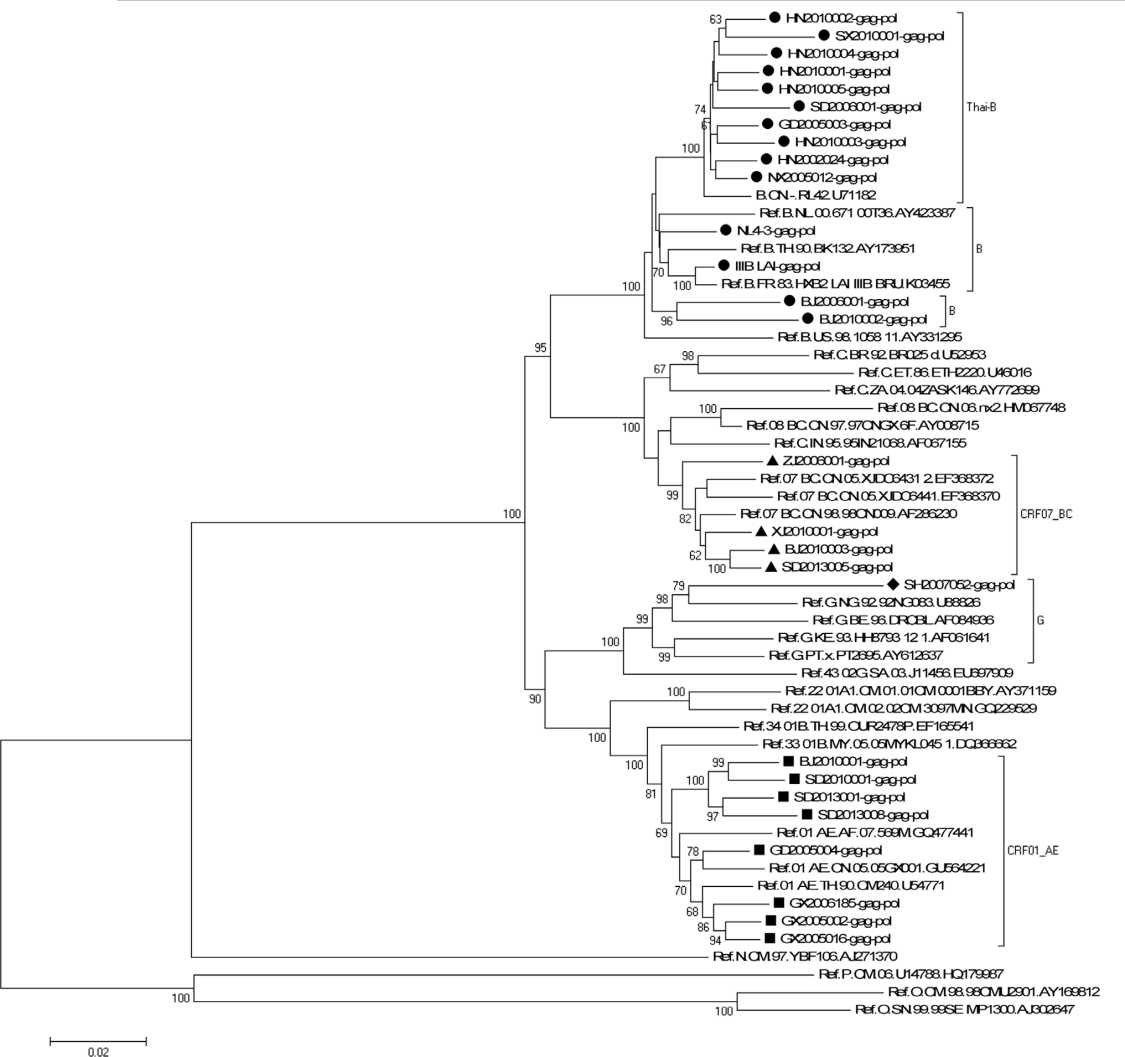
Phylogenetic analysis of characterized HIV-1 *gag-pol* gene sequences. The tree was midpoint rooted. Horizontal branch lengths are drawn to scale (the scale bar represents 0.02 nucleotide substitution per site). Bracket separation is for clarity only. Numbers at the nodes indicate the bootstraps in which the cluster to the right was supported by 60% and higher. Markers in different shapes at the ends of the horizontal branch represent different subtypes to which the sequences belong. Triangle represents the subtype CRF07_BC, square represents the subtype CRF01_AE, rhombus represents the subtype G, and circle represents the subtype B (B represented by black round while Thai-B represented by dark red circle).

**Fig 2:**
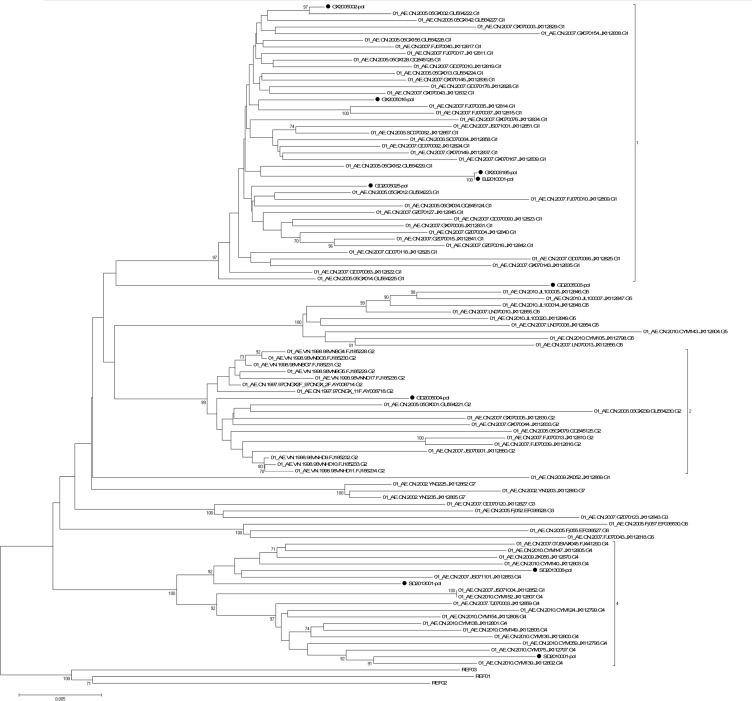
Neighbor-joining tree of HIV-1 CRF01_AE *pol* gene sequences. The phylogenetic tree was constructed with HIV-1 full-length gene *pol* sequences. Horizontal branch lengths are drawn to scale (the scale bar represents 0.005 nucleotide substitution per site). The numbers “1”, “2”, and “4” marked on the bracket separation are clusters that correspond to the clusters in Yi Feng and Xiang He’s study. Numbers at the nodes indicate the bootstraps in which the cluster to the right was supported by 70% and higher. The red circles at the ends of the horizontal branch represent the HIV-1 CRF01_AE samples in our panel. The three reference sequences at the root of the neighbor-joining tree were HIV-1 CRF01_AE sequences from Central Africa. The accession numbers of the three references in the Los Alamos HIV Database are Ref.01_AE.CF.1990.90CF11697.AF197340, Ref.01_AE.CF.1990.90CF4071.AF197341 and Ref.01_AE.CF.1990.90CR402_CAR_E_4003.U51188.

A neighbor-joining tree was constructed to determinate the subtype of each isolate based on 25 full-length *gag-pol* genetic region sequences of the isolates in our program (all the *gag-pol* sequences are available from the Los Alamos Databases and their accession numbers of GenBank are KP178421-KP178445) (GD2005006, GD2005025 and GD2005028 failed in *gag* amplification; GX2006183, SC2009001 failed in *pol* amplification) and 30 standard subtype reference sequences from the Los Alamos HIV sequence database (the reference sequence of subtype Thai-B: B.CN-RL42.U71182 was included) (Fig 1). Subtypes of five other strains (GD2005006, GD2005025, GD2005028, GX2006183 and SC2009001) were designated and identified by REGA Viral Subtyping and the NCBI HIV Subtyping tool based on their gene *gag* sequences (GX2006183-gag: KP178446; SC2009001-gag: KP178447) or gene *pol* sequences (GD2005006-pol: KP178448; GD2005025-pol: KP178449; GD2005028-pol: KP178450). Subtyping assessment results revealed that our panel included four subtypes currently (i.e., B, CRF01_AE, CRF07_BC and G). Thirteen of the 30 samples from this analysis were subtype B (11 strains of Thai-B were included), twelve were subtype CRF01_AE, four were subtype CRF07_BC, and one was subtype G, which was an exceptional subtype in China (Table 2). A neighbor-joining tree of 10 full-length *pol* gene sequences of all 11 CRF01_AE isolates (GX2006183 failed in *pol* amplification) in our program and 93 CRF01_AE full-length *pol* gene sequences in Yi Feng and Xiang He’s study as the reference sequences was constructed to identify the diversity of the CRF01_AE isolates in our panel (Fig 2). In Yi Feng and Xiang He’s study, seven distinct phylogenetic clusters of CRF01_AE were identified. The CRF01_AE isolates in our study were found in three of the seven clusters shown in Fig 2.

**Table 2:** Characterization of expanded viruses.

Sample ID	Infection route	Area of collection	HIV-1 subtype	CD4+T-cells (cells/μl) in plasma	VIDAS HIV p24 II (pg/ml)^a^	Domestic HIV-1 p24 assay(pg/ml)^a^	NucliSENS EasyQ HIV-1 v2.0(million copies/ml)^a^	COBAS Taqman v 2.0(million copies/ml)^a^	Coreceptor predicted by Webpssm	Coreceptor predicted by Geno 2 Pheno (FPR 2.5%)	The top 4 peptides of V3	Number of glycosylation sites in V3 loop	Drug-resistant mutations
GX2005002	Heterosexual sex	Guangxi	CRF01_AE	43	14800	6168	330	252	X4/R5	X4/R5	GPGR	10	NRTI: K70KT
GX2005016	Blood transfusion	Guangxi	CRF01_AE	137	345	74	0.0068	1.07	X4/R5	X4/R5	GPGR	8	None
GX2006183	Unclear	Guangxi	CRF01_AE	293	680	166	0.4	0.892	X4/R5	X4/R5	GLGH	11	None
GX2006185	Heterosexual sex	Guangxi	CRF01_AE	114	371	173	0.15	0.747	X4/R5	X4/R5	GPGH	9	None
GD2005003	Blood transfusion	Guangdong	B	8	11900	6664	1300	152	R5	X4/R5	GPGR	10	None
GD2005004	Heterosexual sex	Guangdong	CRF01_AE	3	1472	498	4.3	8.15	X4/R5	X4/R5	GPGH	9	None
GD2005006	Heterosexual sex	Guangdong	CRF01_AE	2	8630	2372	170	127	X4/R5	X4/R5	GPGQ	11	None
GD2005025	intravenous drug using	Guangdong	CRF01_AE	10	622	270	0.32	1.51	R5	R5	GPGQ	9	PI: K20KT, D30DE, V32EV, M46MR
GD2005028	Blood transfusion	Guangdong	B	36	86	28	2.5	1.16	R5	R5	GPGR	10	PI: V82FIV*
SC2009001	Occupational Exposure	Sichuan	CRF01_AE	503	1390	498	9	10	X4/R5	X4/R5	GPGR	9	None
SX2010001	Blood transfusion	Shanxi	B	76	14000	5742	170	203	R5	R5	GPGR	13	NRTI: M41L*, M184V*, L210W*, T215Y*, K219KN; NNRTI: K103N*, M230L*
SD2010001	Homosexual sex	Shandong	CRF01_AE	1187	22170	7806	43	173	R5	R5	GPGQ	10	None
HN2010001	Blood donation	Henan	B	265	595	314	8.8	4.24	R5	R5	GQGR	13	None
HN2010002	Blood donation	Henan	B	432	14100	5609	130	251	R5	R5	GQGR	11	PI: A71T
BJ2010001	Homosexual sex	Henan	CRF01_AE	1179	3576	909	13	28	R5	R5	GPGQ	10	None
HN2010003	Blood trasfusion	Henan	B	312	7440	3766	180	140	R5	R5	GQGR	13	None
HN2010004	Blood donation	Henan	B	219	3370	1289	32	43.9	R5	R5	GPGQ	13	INI: L68V
BJ2010002	Homosexual sex	Henan	B	1187	101000	92819	5100	2010	R5	R5	GWGR	10	PI: A71T; INI: A128T
XJ2010001	Homosexual sex	Xinjiang	CRF07_BC	1333	18500	7331	1700	1170	R5	R5	GPGQ	9	PI: Q58E
HN2010005	Blood donation	Henan	B	248	18900	12021	860	359	R5	R5	GPGR	9	None
BJ2010003	Homosexual sex	Henan	CRF07_BC	1528	2630	554	250	101	R5	R5	GPGQ	12	INI: H51HQ
NX2005012	Blood transfusion	Ningxia	B	21	237	65	5.8	2.12	R5	R5	GQGR	11	INI: L68V
SH2007052	Heterosexual sex	Shanghai	G	ND	363	50	0.66	0.823	R5	R5	APGQ	10	None
HN2002024	Blood trasfusion	Henan	B	ND	80000	128722	1700	618	X4/R5	X4/R5	GPGR	10	PI: I54M*, L23IL, A71V
SD2013001	Homosexual sex	Shandong	CRF01_AE	197	2575	1047	0.2	22.7	X4/R5	R5	GPGQ	11	INI: P145PS, S153FS
SD2013005	Homosexual sex	Shandong	CRF07_BC	715	107	46	14	3.11	R5	R5	GPGQ	10	None
SD2013008	Homosexual sex	Shandong	CRF01_AE	486	21500	11583	40	254	X4/R5	X4/R5	GPGQ	8	None
BJ2006001	Heterosexual sex	Beijing	B	40	1500	866	4.6	2.49	X4/R5	X4/R5	GRGR	12	NRTI: M41L*, L210W*, T215F*; NNRTI: A98G, K103N*, V179E*, Y181C*, G190A*; PI: L63P, V77I
SD2006001	Blood trasfusion	Shandong	B	74	1600	409	5.8	2.78	X4/R5	X4/R5	GPGR	13	NRTI: M184V*; NNRTI: K103N*; PI: L63P, A71T, V77I, I93L
ZJ2006001	Blood transfusion	Zhejiang	CRF07_BC	38	4060	1488	9.9	17	Unknown	Unknown	Unknown	Unknown	NRTI: K65R*, T69D, K219R; NNRTI: V106M*, Y181C*; PI: L63P, I93L, L10X
IIIB_LAI(Ref)	Unknown	France	B	Unknown	79000	43925	2600	708	X4/R5	X4/R5	GPGR	11	None
NL4-3(Ref)	Recombinant	France	B	Unknown	78000	44829	2200	686	X4/R5	X4/R5	GPGR	12	IN: V151I

ND: Not done, *env*C2V3 PCR was negative.

^a^: Samples for p24 antigen tests(VIDAS HIV p24 II and the domestic HIV-1 p24 assay) and viral load tests(NucliSENS EasyQ HIV-1 v2.0 and COBAS Taqman v 2.0) are culture supernatant of the isolates after expansion.

*: NRTI/NNRTI/PI/INI Major drug-resistance mutations. Mutations without “*” are minor resistance mutations, accessory mutations or single nucleotide polymorphisms (SNP).

Ref: Reference virus. IIIB_LAI and NL4-3 are reference virus with clear background information and high replication capacity but are not primary isolates.

The expanded virus was characterized in terms of infection route, area of origin, CD4+T cells concentration in blood sample, viral RNA concentration (Roche TaqMan v2.0 and BioMerieux NucliSENS EasyQ HIV-1 v2.0), p24 concentration (BioMerieux VIDAS HIV p24 II and One domestic HIV-1 p24 antigen assay), coreceptor usage (Webpssm and Geno 2 Pheno (FPR 2.5%)), the four identical residues at the tip of v3 loop, number of glycosylation sites in v3-loop, and drug-resistance mutations in *pol*.

### The prediction of co-receptor usage, the four amino acids at the tip of the V3-loop and the glycosylation sites by *env*V3 sequences of each isolate

The predominant co-receptor usage of each of the virus isolates was determined based on genotype analysis by two online software programs: WebPSSM and Geno2Pheno (G2P) prediction (Table 2). Twenty-nine *env*C2V3 sequences (KP178451-KP178479) of the thirty samples were amplified successfully. Fifteen of the thirty samples tested exhibited a CCR5 phenotype while fourteen of the thirty samples test exhibited a CXCR4/CCR5 phenotype in both assays (the *env*C2V3 amplification of ZJ2006001 was negative). However, the predictions for GD2005003 and SD2013001 were opposite according to the two assays (Table 2). The four amino acids at the tip of the V3-loop and the number of glycosylation sites on the V3-loop for each isolate are shown in Table 2.

### The determination of viral drug resistance mutations

Online tools at the Stanford University HIV-1 database (http://hivdb.stanfrod.edu/hiv/) were used to determine the drug-resistance mutations in the full-length sequences of the gene *pol* of each isolate. Their tolerance for each kind of antiviral drug related to the mutations were also predicted to consummate the fully characterization of the isolates (Table 2).

### Statistical analysis of the p24 concentrations and viral loads of the HIV-1 isolates

P24 antigen concentrations were quantified using a BioMerieux VIDAS HIV p24 II assay and a domestic p24 test assay. No isolates were below the lower limits of the assays (3.0 pg/ml for the VIDAS HIV p24 II assay and 5.0 pg/ml for the domestic p24 test assay). The results of the p24 concentration test by the BioMerieux VIDAS HIV p24 II assay ranged from 86.1 pg/ml to 1.01×10^5^ pg/ml. The results of the p24 concentration test by the domestic p24 test assay ranged from 28.3 pg/ml to 1.29×10^5^ pg/ml (Table 2). The viral loads were measured by the Cobas AmpliPrep/Cobas TaqMan HIV-1 test version 2.0(CAP/CTM v2.0, Roche) and the NucliSens EasyQ HIV-1 version 2.0 (EasyQ v2.0, BioMerieux). No isolates were below the lower limits of the assays (5.747 IU/ml for the EasyQ v2.0 assay and 34 IU/ml for the CAP/CTM v2.0 assay). The viral load test by CAP/CTM v2.0 ranged from 7.47×10^5^ copies/ml to 2.01×10^9^ pg/ml. The viral load test with the EasyQ v2.0 ranged from 6.8×10^3^ copies/ml to 5.1×10^9^ copies/ml (Table 2).

**Fig 3:**
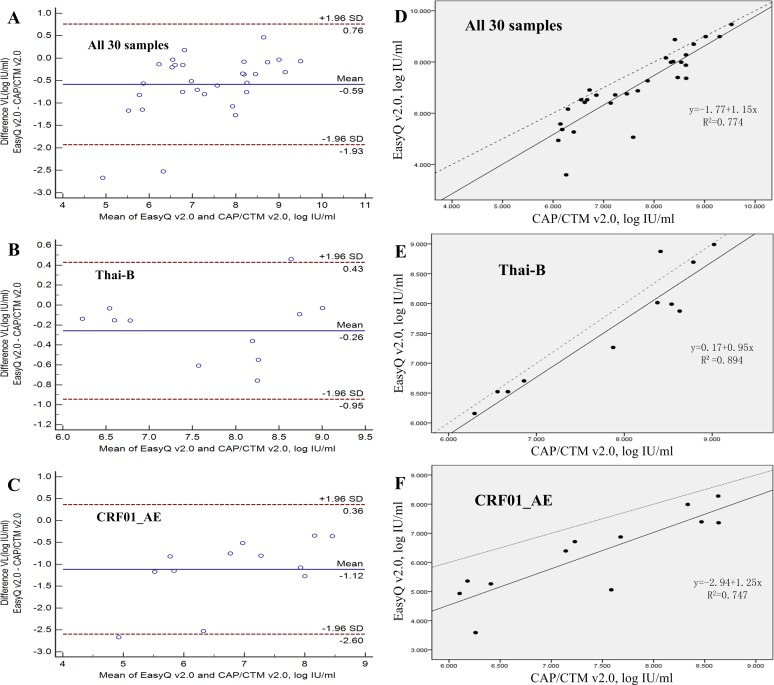
Agreement and linear relationship between the Cobas AmpliPrep/Cobas TaqMan HIV-1 test version 2.0 (CAP/CTM v2.0) and the NucliSens EasyQ HIV-1 version 2.0 (EasyQ v2.0) calculated using the Bland-Altman model. (A) Agreement between EasyQ v2.0 and CAP/CTM v2.0 when used to measure all 30 samples in the panel. (B) Agreement between EasyQ v2.0 and CAP/CTM v2.0 when used to measure 11 clade Thai-B samples in the panel. (C) Agreement between EasyQ v2.0 and CAP/CTM v2.0 when used to measure 12 clade CRF01_AE samples in the panel. For A, B and C, solid horizontal lines indicate the mean values, and dashed horizontal lines indicate the +1.96SD and _1.96SD values. (D) The linear relationship between the CAP/CTM v2.0 and EasyQ v2.0 when used to measure all 30 samples. (E) The linear relationship between the CAP/CTM v2.0 and EasyQ v2.0 when used to measure 11 clade Thai-B samples. (F) The linear relationship between the CAP/CTM v2.0 and EasyQ v2.0 when used to measure 12 clade CRF01_AE samples. For D, E and F, solid line represents the fitted regression line and the dashed line represents the equality line.

As for the two assays providing viral load tests, according to the results of the performance, EasyQ v2.0 showed a significant linear correlation (R = 0.880, p<0.001) and high agreement (93.33%, 28/30) with CAP/CTM v2.0 for all 30 samples (Table 3 and Fig 3A). The regression equation used for fitting was: EasyQ v2.0 = -1.77+1.15×CAP/CTM V2.0 (Fig 3D). The mean difference between the quantitative values measured by EasyQ v2.0 and CAP/CTM v2.0 was 0.587 log IU/ml (SD = 0.687; Table 3). For the different subtype isolates (subtype Thai-B and subtype CRF01_AE, Table 3), a significant linear correlation (R = 0.946 for subtype Thai-B, p <0.001; R = 0.864 for subtype CRF01_AE, p<0.001; Table 3) and high agreement (90.91% for Thai-B and 91.67% for CRF01_AE; Fig 3) were observed. The mean difference between the values measured by EasyQ v2.0 and CAP/CTM v2.0 was 0.260 log IU/ml (SD = 0.350) for subtype Thai-B (p = 0.260) and 1.120 log IU/ml (SD = 0.756) for subtype CRF01_AE (p<0.001). The number of samples with the quantitative differences between EasyQ v2.0 and CAP/CTM v2.0 exceeded 0.5 log IU/ml varied from each subtype: three samples (27.27%) for subtype Thai-B and 10 samples (83.33%) for subtype CRF01_AE. Furthermore, six subtype CRF01_AE samples showed quantitative differences of >1 log IU/ml, while no samples of Thai-B showed quantitative differences of >1 log IU/ml (Table 3).

**Table 3:** Comparison of the EasyQ v2.0 and CAP/CTM v2.0 using samples belonging to different clades.

			Paired t-test	Pearson correlation test	Bland-Altman model		Outliers(n[%])	
	n	Difference in viral load(log IU/ml)^a^	Sig^b^	R value^c^[p-value]	Limits of agreement	Agreement(n/N[%])	>0.5 log IU/ml	>1 log IU/ml
Total	30	0.587±0.687	<0.001	0.880[<0.001]	-1.93,0.76	28[93.33]	15[50.00]	6[20.00]
Thai-B	11	0.260±0.350	0.026	0.946[<0.001]	-0.95,0.43	10[90.91]	3[27.27]	0[0]
CRF01_AE	12	1.120±0.756	<0.001	0.864[<0.001]	-2.60,0.36	11[91.67]	10[83.33]	6[50.00]

EasyQ v2.0: NucliSens EasyQ HIV-1 version 2.0; CAP/CTMv2.0: Cobas AmpliPrep/Cobas TaqMan HIV-1 test version 2.0.

^a^: Values are expressed as mean±SD.

^b^: Significance calculated using paired t-test (p-values).

^c^: Correlation coefficient (R value) calculated using Pearson correlation test.

**Fig 4:**
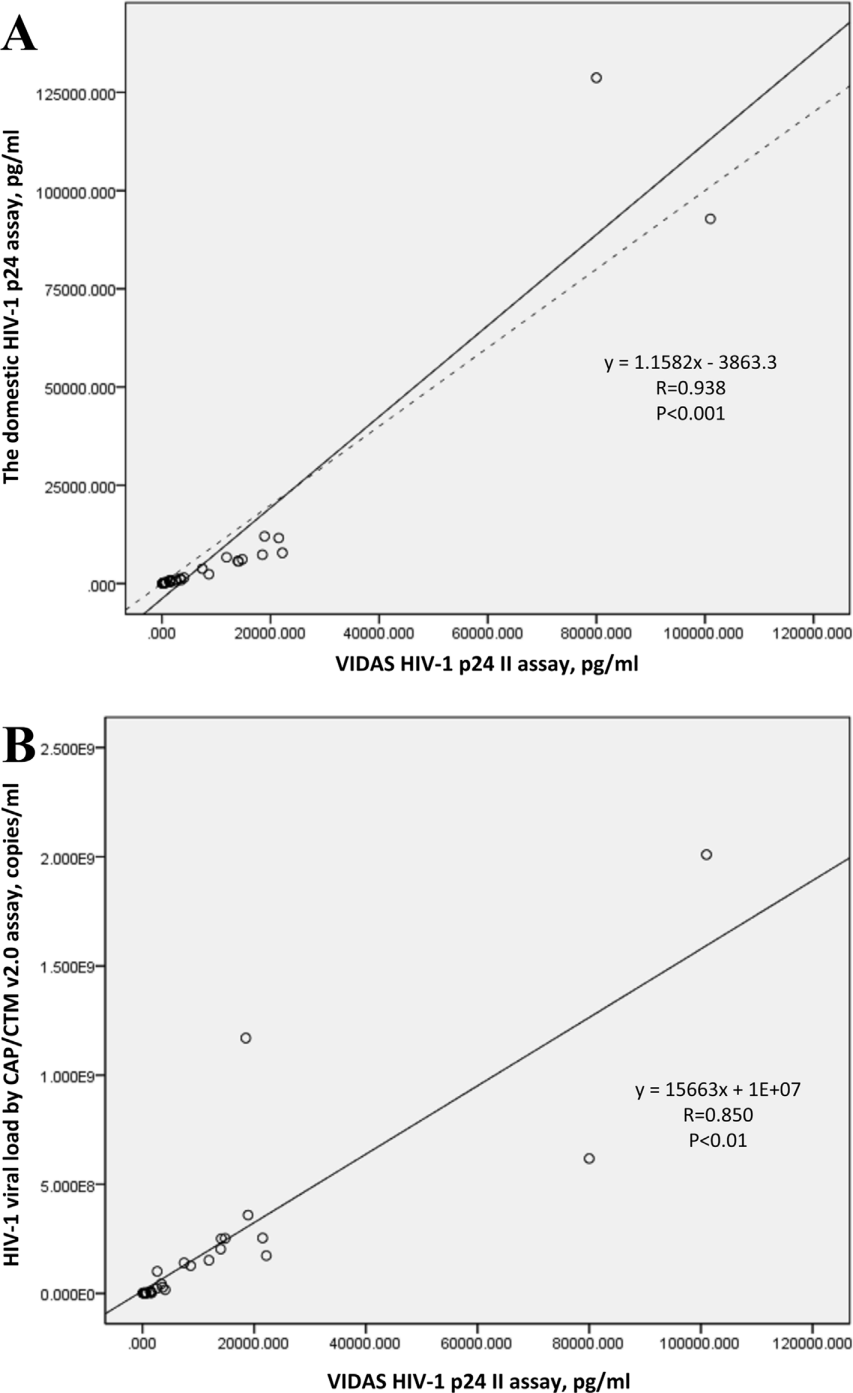
The linear relationship between the two assays of HIV-1 p24 measurement and the linear relationship between p24 antigen concentration and HIV-1 viral load based on the values of all 30 samples. (A) The linear relationship between the BioMerieux VIDAS HIV p24 II assay and the domestic HIV-1 p24 assay when used to measure all 30 samples. Solid line represents the fitted regression line and the dashed line represents the equality line. (B) The linear relationship between HIV-1 p24 antigen concentration and viral load based on all 30 isolates, tested by BioMerieux VIDAS HIV p24 II and Roche COBAS Taqman version 2.0, respectively. Solid line represents the fitted regression line.

As for the two assays of p24 antigen tests, the Pearson correlation coefficient for the two HIV-1 P24 antigen assay based on all 30 samples in the panel was 0.938 (p<0.01). The correlation coefficient for the p24 concentrations tested by the BioMerieux VIDAS HIV p24 II assay and the viral loads tested by the Roche COBAS Taqman version 2.0 assay based on all the 30 isolates was 0.850 (p<0.01; Fig 4).

## Discussion

With the diversity of HIV-1 increasing, HIV-1 isolates of each subtype spread broader and faster around the world. The EQAPOL program was established by the NIH/NIAID and other cooperative institutions and their efforts were aimed at the development of an HIV subtype panel that encompassed the genetic and geographic diversity currently present worldwide. Accordingly, we developed an HIV-1 isolate panel that can represent the diversity of HIV-1 strains circulating in China. This panel will help with the evaluation of HIV surveillance or diagnostic assays and research on HIV vaccines or antiviral drugs in China [13]. In our study, blood samples from HIV infected people in China were collected from 2002 to 2013. 30 HIV-1 primary strains of high-titer and high-volume were obtained successfully. These isolates were all well preserved in liquid nitrogen and can be used as the standard substances or samples in evaluation of HIV-1 assays, antiviral drug screening or vaccine development conveniently. The subtypes of these isolates not only include China’s frequent subtype B, CRF01_AEand CRF07_BC, but also a strain of subtype G which was infrequent in China was isolated from a plasma sample from Shanghai. Shown by Yi Feng and Xiang He’s research, the CRF01_AE epidemic in China is remarkably complex [35]. In their study, seven distinct phylogenetic clusters of CRF01_AE were identified. The CRF01_AE isolates in our study were found in three of the seven clusters shown in Fig 2, which means the CRF01_AE isolates in our panel can represent the current complex diversity of this subtype to some extent. We amplified and sequenced the fundamental genes; i.e., *gag*, *pol*, *env*C2V3 for each isolate. The co-recepter usage, glycosylation sites, four residues at the tip of the v3-loop for each isolate, also the drug-resistance mutations on gene *pol* were predicted to consummate the characterization of the isolates in this panel. This accurate and comprehensive characterization will make the panel convenient to use in the future.

The comparison of the p24 or viral load assays on the isolates illustrated how this panel may be used for evaluation of HIV-1 diagnostic assay performance. For example, according to Fig 3, compared with CAP/CTM v2.0, the EasyQ v2.0 showed a greater variation in the values measured for CRF01_AE samples than those for Thai-B samples. By Bland-Altman analysis, we could see that the distance between the mean values of the different results from the two assays in the viral loads of subtype CRF01_AE samples, which are shown as solid horizontal lines in Fig 3C, and the origin, which is shown as the zero point in Fig 3C (1.120), were much farther than that of Thai-B samples (0.260; Fig 3B). Furthermore, the proportion of subtype Thai-B samples showing quantitative differences between the two assays >0.5 log IU/ml or 1 log IU/ml was significantly lower than that for subtype CRF01_AE (Table 3). These data suggest higher levels of agreement between these two assays when measuring Thai-B samples than for CRF01_AE samples. A similar conclusion was shown in Sihong Xu’s study [36]. Higher values measured by CAP/CTM v2.0 may because of the fact that CAP/CTM v2.0 reduces under-quantification of the HIV-1 viral load by using two dual-labeled hybridization probes targeting both the *gag* and LTR regions, while EasyQ v2.0 targeted the HIV-1 *gag* gene only. The performance of the automated extractor of CAP/CTM v2.0 may also contribute to the higher values and stable performance of CAP/CTM v2.0. The lower agreement between the two assays when measuring subtype CRF01_AE samples may also due to different hybridization probes, which indicates that the probe targets in the sequence may not be conservative enough in subtype Thai-B and CRF01_AE, and at least one of the assays under-quantified the viral loads of subtype CRF01_AE samples. To face this problem, it is strongly suggested that the same version of the same viral load assay be used consistently during clinical treatment. Much more importantly, the genetic diversity of HIV-1 (including the genetic variability within HIV-1 strains of the same subtype) must be taken into account when designing primers and probes for HIV-1 viral load assays. Another example is the two different results for co-receptor usage prediction of GD2005003 and SD2013001 (Table 2) may be caused by the different calculation principles of the two software programs; i.e., Geno2pheno and PSSM, or the different FPR values chosen for Geno2pheno.

The entry of HIV-1 into host cells is mediated by interactions between the virus envelope (i.e., *env*) glycoprotein (gp120/gp41) and host-cell receptor [38]. The third hypervariable region 3 (V3) of the HIV-1 gp120 protein consists of 35 amino acids and plays an important role in viral infection by promoting the interaction between the virus and its co-receptor in the host cell membrane [39]. N-glycan represent approximately 50% of the molecular mass of gp120 and serves as a potential antigenic determinant and a shield against immune recognition [40]. It is convinced that HIV-1 escape from neutralizing antibodies through the extensive variability of the viral envelope glycoproteins, especially gp120 while the four amino acids at the tip of the V3 loop are subjected to strong purifying selection pressure due to their functional importance [41, 42]. Therefore, using the consensus sequence on *env*C2V3 to predict the co-receptor usage of each isolate, glycosylation sites on the v3-loop and identification of the four residues at the tip of the V3 loop is necessary for infection relevant experiments and studies.

In conclusion, by isolating, expanding, sequencing and analyzing the HIV-1 strains from blood samples of HIV-1 infected persons, our study produced a panel of HIV-1 primary strains of a variety of HIV-1 subtypes circulating in China with clear and complete fundamental sequences and biological features. Our program will continue to collect HIV-1 strains for more subtypes to develop a more comprehensive panel that can represent the diversity, the current dynamic and complex epidemic of HIV-1 more completely and accurately. Especially for samples of CRF08_BC, because it is one of the frequent subtypes circulating in China besides CRF07_BC, CRF01_AE and B. Also, more effort should be spent on the isolation of viruses from the acute/early stage of infection to analyze the transmitted or early founder viruses. This is an important step in achieving a molecular understanding of HIV-1 transmission and for potentially developing an effective HIV/AIDS vaccine since direct analysis of HIV-1 at or near the moment of transmission is practically impossible [43]. In our study, we created a basic protocol and standard for sample collection, inclusion and analysis by developing the panel. Under the circumstance of high variable HIV-1 strains of each subtype spread throughout the world, the creation of this panel will not only serves as the standard for assays, vaccines and drugs development andevaluation, but also as the samples for diversity related HIV-1 research to help improve the current situation when many researches on HIV-1 diversity still stay on the genome sequence level. By offering HIV-1 isolates isolated from blood samples directly, researches based on the panel will have high simulation and high reproducibility when compared with researches based on infectious clones of the viruses. In this way, our panel may also play a role in antiviral drug screening and vaccine development.
